# Generalized granuloma annulare associated with essential thrombocythemia

**DOI:** 10.1002/ccr3.2812

**Published:** 2020-03-23

**Authors:** Sai P. Polineni, Steven Lawyer

**Affiliations:** ^1^ University of Miami Miller School of Medicine Miami Florida; ^2^ Good Health Clinic Tavernier Florida

**Keywords:** essential thrombocythemia, generalized granuloma annulare, JAK2, janus kinase 2, myeloproliferative neoplasm

## Abstract

The only myeloproliferative neoplasm associated with generalized granuloma annulare (GA) is chronic myelogenous leukemia (CML). We present the first reported case of GA in a patient with essential thrombocythemia (ET). Future work investigating the shared pathophysiology of GA‐associated CML and ET may improve our understanding of GA pathophysiology and treatment.

## Graphical abstract

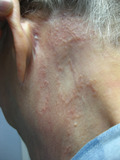

## 1 INTRODUCTION

Generalized granuloma annulare (GA) is a granulomatous skin disorder of unknown pathophysiology and course. While GA is associated with multiple systemic disorders, associations between GA and myeloproliferative neoplasms are rarely reported. This is the first reported case of GA in a patient with essential thrombocythemia.

Generalized granuloma annulare (GA) is a granulomatous cutaneous reaction of unknown cause, variable course, and multiple associations with other diseases.^1^ Case reports have described associations between GA and a variety of lymphomas, leukemias, and visceral malignancies.^1^ Among myeloproliferative neoplasms, chronic myelogenous leukemia (CML) has been reported to be associated with GA in a select few case reports.^2^, ^3^ However, no association has ever been found between GA and other types of myeloproliferative neoplasms. Herein, we present the first reported case of generalized granuloma annulare in a patient with long‐standing essential thrombocythemia (ET), a type of myeloproliferative neoplasm.

## 2 CASE REPORT

A 61‐year‐old Caucasian female with a 10‐year history of JAK2‐positive ET presented to our charity care clinic for a wellness visit and evaluation of a skin rash that has persisted for 2 years. Medical history was significant for adult onset asthma at age 45, chronic anxiety, dyspnea, and hypertension. Daily medication use consisted of aspirin, hydroxyurea, valsartan, and venlafaxine. The patient had also been prescribed albuterol sulfate and a fluticasone and salmeterol oral inhaler to be used as needed. Laboratory data were as follows: 4.96 K/µL WBCs with normal differential, 12.8 g/dL hemoglobin, 340 k/µL platelets, and 102.5 M/µL MCV. Serum chemistry studies and urinalysis were within normal limits.

**Figure 1:**
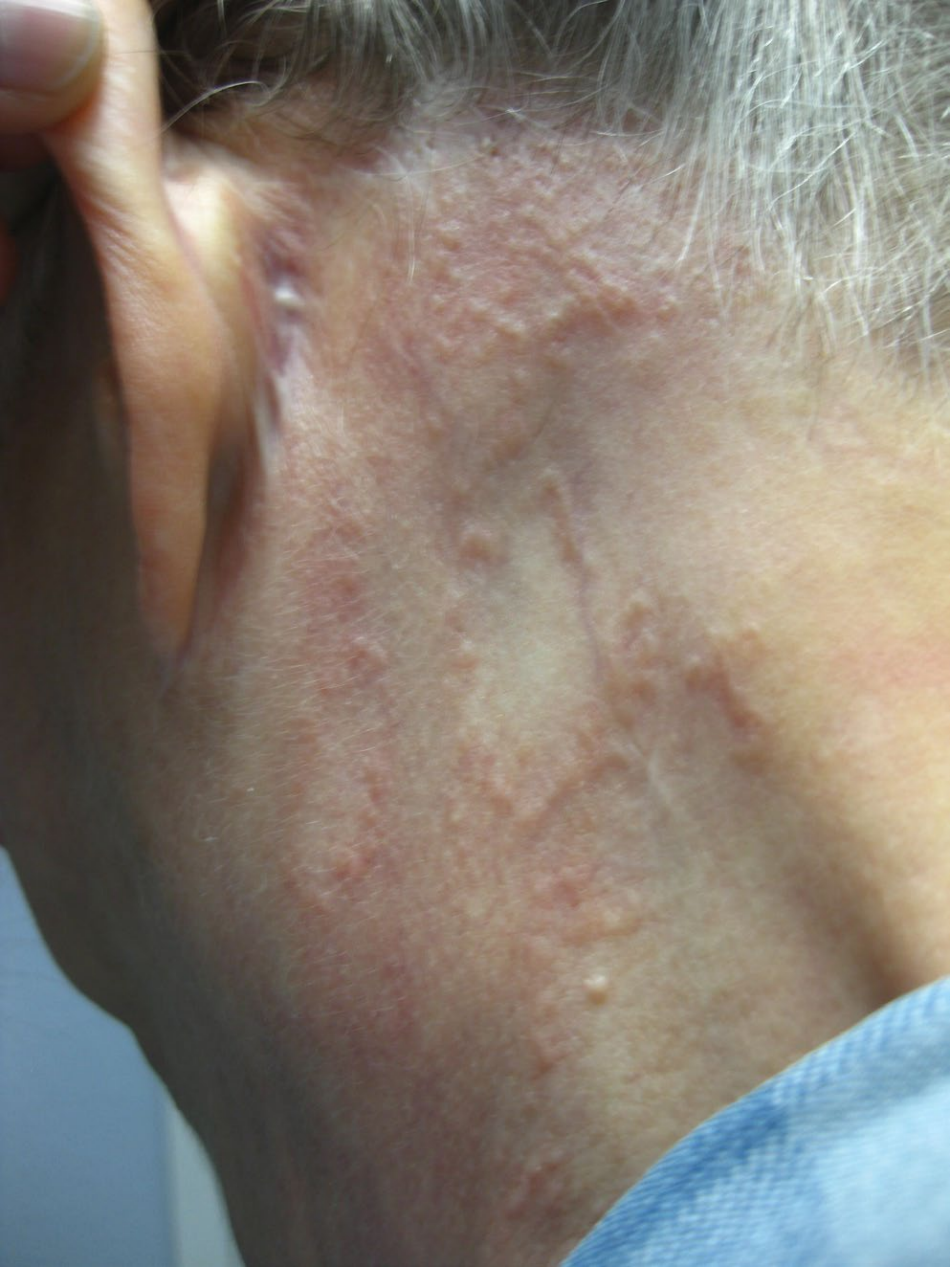
Presentation of generalized GA at the left postauricular region

**Figure 2:**
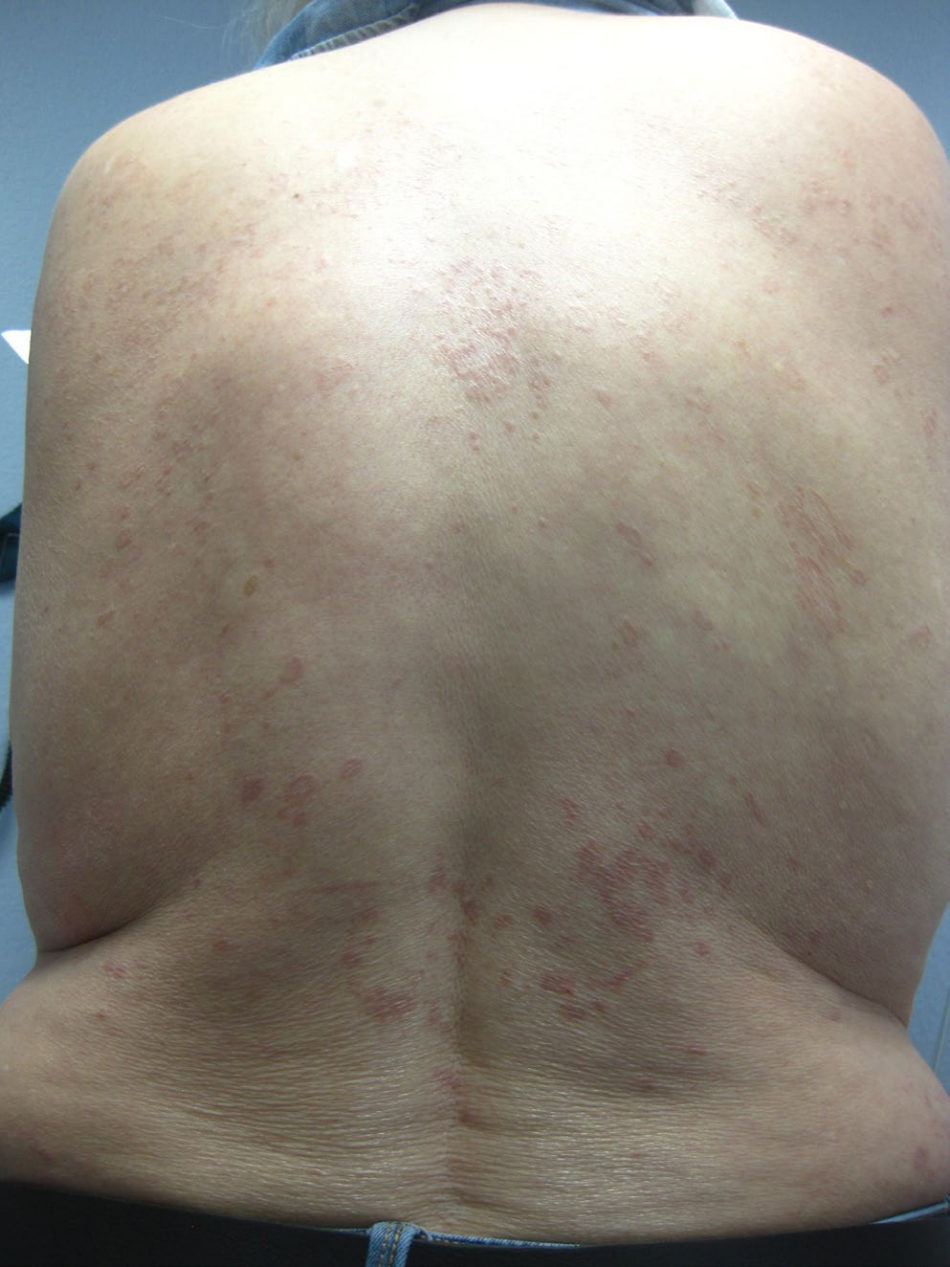
Presentation of generalized GA at the back

**Figure 3:**
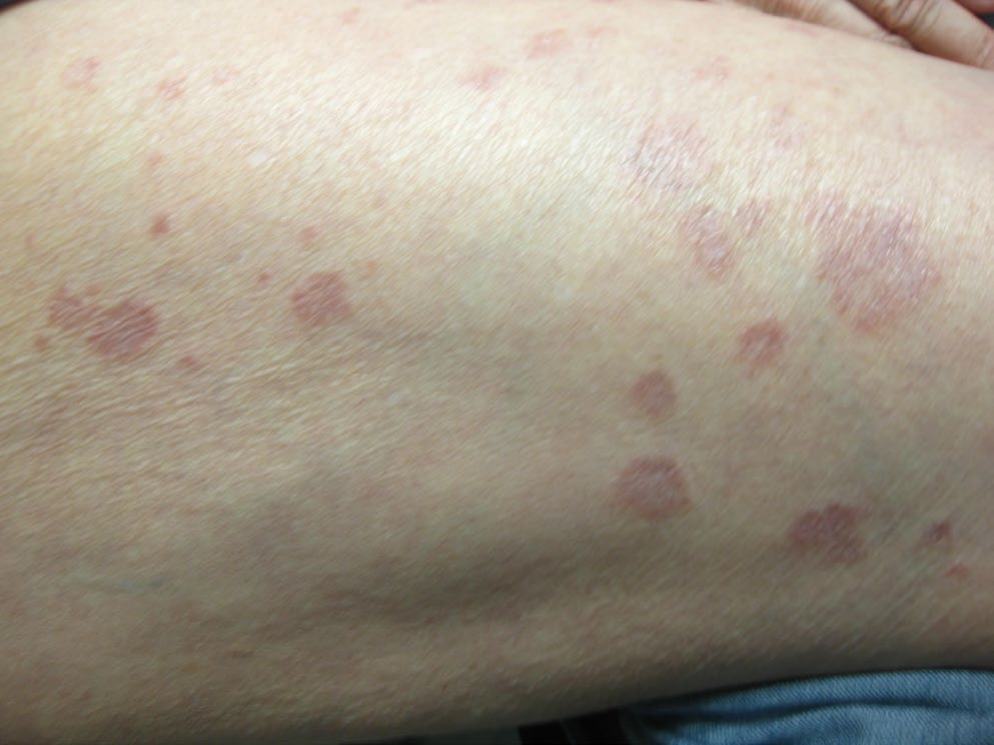
Presentation of generalized GA at the right lateral thigh

Histopathologic examination of her skin lesions revealed palisading histiocytes and lymphocytic infiltrates with neutrophils and eosinophils localized to perivascular spaces and the periphery of granulomatous areas within the papillary dermis, confirming generalized GA. Initial presentation of the GA was reported as nonpruritic elliptical shapes on her left forearm and a nonpruritic pink circular lesion on the left anterolateral neck that were initially presumed to be dermatophytosis. Since initial presentation, the rash has developed into diffusely scattered annular or linear lesions with central paling and raised, erythematous borders that now affect the entire body, sparing only the face, scalp, palms, and soles (Figures 1, 2, 3). Since diagnosis, treatment with topical and systemic steroids and PUVA showed no improvement at follow‐up and socioeconomic issues have temporarily prevented further treatment with more novel therapies.

## 3 DISCUSSION

Generalized GA has been shown to occur in the setting of lymphomas, leukemias, myelodysplasias, and visceral malignancies, though the temporal relationship between presentation of GA and of the malignancy has not always been constant.^2^, ^3^, ^4^, ^5^ It has also been associated with diabetes, thyroid disease, systemic lupus erythematosus, and other autoimmune conditions.^4^, ^5^, ^6^ However, attempts to determine the pathophysiology of the disease or to evaluate the association between leukemias and GA have not yielded conclusive information. Cell‐mediated delayed hypersensitivity to a yet unknown antigen, which results in the production of immunologically active cytokines and related molecules, is one potential explanation for the granulomatous inflammation and skin eruptions seen in generalized GA.^7^ While eosinophils have often been found in histopathologic studies of generalized GA, their relegation to the periphery of granulomatous lesions and the lack of association between the number of eosinophils—or their absence altogether—and the type and symptomatology of GA suggest that the pathophysiology of GA does not significantly involve eosinophilic activation.^6^, ^8^, ^9^ Similar variability is seen both across and within GA subtypes in the depth to which GA affects the dermis dermal depth. While many cases of generalized GA involve both the papillary and reticular dermis, the predominance of the disease to the papillary dermis visualized in our patient is not an uncommon histologic presentation.^8^

Generalized GA is primarily treated via phototherapy rather than oral steroids, though efficacy is limited by a high recurrence rate following treatment cessation.^7^ Furthermore, generalized GA tends to have a poorer response to therapy than other types of GA and necessitates a better understanding of the underlying pathophysiology of the disease.^7^ Given the low incidence and prevalence of generalized GA, medication trials are often relegated to the realm of case reports and series rather than large‐scale clinical trials. Thus, efforts to understand and treat this condition may require a retrograde approach: evaluating successful treatments and associated diseases in order to backtrace the pathophysiology of the disease. Published work reporting the success of methotrexate, apremilast, calcineurin inhibitors, and fumaric acid esters, among others, in treating generalized GA serve to guide us in this fashion.

In the literature, the presentation of generalized GA coinciding with CML has rarely been reported and coincidence with ET has never been reported. Thus, while incidence and prevalence of such an occurrence are low, the potential for generalized GA to be associated with a pathophysiologic mechanism shared between CML and ET, both of which are myeloproliferative neoplasms, represents an avenue through which further understanding of the pathophysiology of generalized GA may be gained. Of note, our patient was diagnosed with JAK2‐positive ET, a feature of ET that rarely occurs in CML patients.^10^ Recent work investigating the immune pathogenesis of GA has identified significantly increased JAK and Th2 signaling in the pathophysiology of GA, highlighting the JAK pathway as a potential target for drug therapy.^11^ A subsequent study using tofacitinib, a JAK inhibitor, in a patient with generalized GA yielded significant improvement in the Granuloma Annulare Scoring Index score.^12^ Furthermore, immunohistochemistry revealed that JAK‐STAT signaling was found to be constitutively activated in lesional tissue, but not in peripheral blood cells.^12^ These studies on the potential use of JAK‐inhibitors help shed further light on the biological development of generalized GA and of granulomatous diseases as a whole.^13^

Whether generalized GA is associated with a shared characteristic of CML and ET or with JAK‐2 positive myeloproliferative neoplasms, this present case constitutes the first reported case of generalized GA in a patient with ET, expanding the list of diseases known to be associated with generalized GA. Furthermore, it suggests that CML is not the only myeloproliferative neoplasm associated with generalized GA and that future work should focus on the viability of JAK‐inhibitors to both explain and treat the pathologic mechanisms underlying generalized GA.

## CONFLICT OF INTEREST

None declared.

## AUTHOR CONTRIBUTIONS

SP: collected data and drafted manuscript. SL: patient follow‐up and edited manuscript.
